# Supplementary material for: Redox regulation of G protein oligomerization and signaling by the glutaredoxin WG1 controls grain size in rice
Source: EMBO J. 2025 May 19;44(13):3742–63. doi: 10.1038/s44318-025-00462-9 (PMC12216599; doi:10.1038/s44318-025-00462-9)
Supplement: Supplementary file 1 — Appendix [file 44318_2025_462_MOESM1_ESM.pdf]

Appendix for "Redox regulation of G protein oligomerization and signaling by the glutaredoxin WG1 controls grain size in rice"

Table of contents

Appendix Figure S1.........Page 2

Appendix Table S1.........Pages 3-4

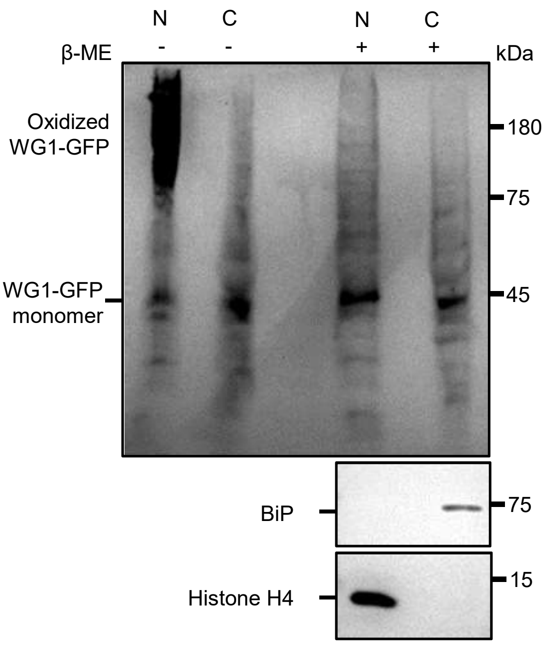

Appendix Figure S1. Subcellular fractionation and immunoblot assays using reducing and non-reducing gels.

10-day old young seedlings of *proWG1:WG1-GFP* plants were used to isolate the nuclear protein fraction (N) and the cytoplasmic protein fraction (C). WG1-GFP proteins in both fractions were analyzed using reducing (with 33% β-ME) and non-reducing gels immunoblotted with an antibody against GFP. BiP, a luminal-binding protein, was used as cytoplasmic marker. Histone H4 was used as nuclear marker.

Appendix Table S1. List of primers used in this study.

	Primers for constructs
Construct name	Primers sequences (F: Forward R: Reverse)
SK-gRNA-GS3N	F: ggcaCCGGCGCCGCCCGACCCATG
	R: aaacCATGGGTCGGGCGGCGCCGG
pro35S:MYC-WG1	F: cgaaatcgatggatcctaATGCAGTACGGAGCGGCGGC
	R: gagctcactagttaattaaCTAGAGCCAGAGCGCGCC
pro35S:MYC-$WG1^{C46,49S}$	F: ctgaagaggacttgaattcggtacccATGCAGTACGGAGCGGCG
	R: aagacgcgtcctaggctacgtaggatccaCTAGAGCCAGAGCGCGCCGG
pro35S:GFP-GS3	F: tgaactatacaaaggcgcgccaATGGCAATGGCGGCGGCG
	R: ctctagaactagttaattaaTCACAAGCAGGGGGGGCAGC
AD-GS3	F: gccatggaggccagtgaattcATGGCAATGGCGGCGGCG
	R: attcatctgcagctcgagctcgTCACAAGCAGGGGGGGCAGC
AD-$GS3^{1-94}$	F: gccatggaggccagtgaattcATGGCAATGGCGGCGGCGCC
	R: attcatctgcagctcgagctcgTCATCTGCACAAACAGCGAAACT
AD-$GS3^{95-232}$	F: gccatggaggccagtgaattcGCAAGTGCGTGCTGCCTCAGCTA
	R: attcatctgcagctcgagctcgTCACAAGCAGGGGGGGCAGCA
AD-$WG1^{C46,49S}$	F: gccatggaggccagtgaattcATGCAGTACGGAGCGGCG
	R: attcatctgcagctcgagctcgCTAGAGCCAGAGCGCGCCG
	G1M-F:CGTGAGACATGCAAGAGCTGCTCACGCTGAACACCACCAC
	G1M-R: CAGCTCTTGCATGTCTCACGCCGTGAAGCGCCTCTTCTGC
BD-GS3	F: aggacctgcatatggccatggagATGGCAATGGCGGCGGCG
	R: ctagttatgcggccgctgcaggTCACAAGCAGGGGGGGCAGC
cYFP-GS3	F:TGACTATGCGTCGACATATGAGCTCATGGCAATGGCGGCGGCGCC
	R: agtcactatggtcgaTCACAAGCAGGGGGGGCAGC
	YC3-F: cacgggggactctag ATGGCCGACAAGCAGAAGAAC
	YC3-R: GAGCTCATATGTCGACGCATAGTCAGGAACATCGT
nYFP-GS3	F: gaggaagagtatatgcctatggaaATGGCAATGGCGGCGGCG
	R: agtcactatggtcgaTCACAAGCAGGGGGGGCAGC
	YN3-F: cacgggggactctagATGGTGAGCAAGGGCGAGGAG
	YN3-R: ttccataggcatatactcttcctc
nYFP-WG1	F: gaggaagagtatatgcctatggaaATGCAGTACGGAGCGGCG
	R: agtcactatggtcgaCTAGAGCCAGAGCGCGCCG
	YN1F: cacgggggactctagATGGTGAGCAAGGGCGAGGAG
	YN1R: ttccataggcatatactcttcctc
MBP-GS3	F: agggaaggatttcagaattcATGGCAATGGCGGCGGCGCC
	R: agtgccaagcttgcctgcagTCACAAGCAGGGGGGGCAGC
MBP-FLAG	F: GATCCgactacaaggacgacgatgacaaaTGAA
	R: AGCTTTCAtttgtcatcgtcgtccttgtagtcG
GST-RGB1	F: atctggttccgcgtggatccATGGCGTCCGTGGCGGAGCT
	R: agtcagtcacgatgcggccgctcTCAAACTATTTTCCGGTGTC
cLUC-GS3	F: ggggcggtacccggggatccATGGCAATGGCGGCGGCG
	R: cgaaagctctgcaggtcgacTCACAAGCAGGGGGGGCAGC
GS3-nLUC	F: gagctcggtacccggggatccATGGCAATGGCGGCGGCG
	R: gcgtacgagatctggtcgacCAAGCAGGGGGGGCAGCA
cLUC-EOG1	F: ggggcggtacccggggatccATGGCGTCGTCGGCGGAG

	R: cgaaagctctgcaggtcgacTTAGGTCGCTTCAGCAGTCG
RGB1-nLUC	F: gagctcggtacccggggatccATGGCGTCCGTGGCGGAGCT
	R: gcgtacgagatctggtcgacAACTATTTTCCGGTGTC
FLAG-GS3-4	F: gttccagattacgctggatccgaattcATGGCAATGGCGGCGGCG
	R: agtggtggtggtggtggtgctcgagACGCCGCCCCACATGAGGA
	Fl-S3-4M-F: TGCTCATCCTCTCCTCCTCCTTCAACCTCAAGAGGCCGAG
	Fl-S3-4M-R: AAGGAGGAGGAGAGGATGAGCAGCCGCCGGCGGCGCTGCT
FLAG-GS3-2	F: gttccagattacgctggatccgaattcATGGCAATGGCGGCGGCG
	R: agtggtggtggtggtggtgctcgagCAAGCAGGGGGGGCAGCA
FLAG-GS3	F: aaggacgatgacgataagttcgaaATGGCAATGGCGGCGGCG
	R: ctgcaggtcgacactagtTCACAAGCAGGGGGGGCAGC
GS3-4-FLAG	F: ggggacgagctcggtaccATGGCAATGGCGGCGGCG
	R: gtctttgtagtcttcgaaACGCCGCCCCACATGAGGA
GS3-2-FLAG	F: ggggacgagctcggtaccATGGCAATGGCGGCGGCG
	R: gtctttgtagtcttcgaaCAAGCAGGGGGGGCAGCA
FLAG-WG1^{C46,49S}	F: gttccagattacgctggatccgaattcATGCAGTACGGAGCGGCG
	R: agtggtggtggtggtggtgctcgagGAGCCAGAGCGCGCCGGCCT
Primers for identification of mutants	
Mutant name	Primers (F: Forward R: Reverse)
gs3-c1	F: GCCGCCGGCGCCGCCCGGCC
	R: TTTTGAAGCAAGATCGAAGGA
Primers for quantitative RT-PCR	
Gene name	Primers (F: Forward R: Reverse)
ACTIN1	F: TGCTATGTACGTCGCCATCCAG
	R: AATGAGTAACCACGCTCCGTCA
WG1	F: AGCAGGCGTGGTACATG
	R: TTCACGGCGTGGCACAT
