# Supplementary material for: Redox regulation of G protein oligomerization and signaling by the glutaredoxin WG1 controls grain size in rice
Source: EMBO J. 2025 May 19;44(13):3742–63. doi: 10.1038/s44318-025-00462-9 (PMC12216599; doi:10.1038/s44318-025-00462-9)
Supplement: Supplementary file 8 — Expanded View Figures [file 44318_2025_462_MOESM8_ESM.pdf]

Expanded View Figures

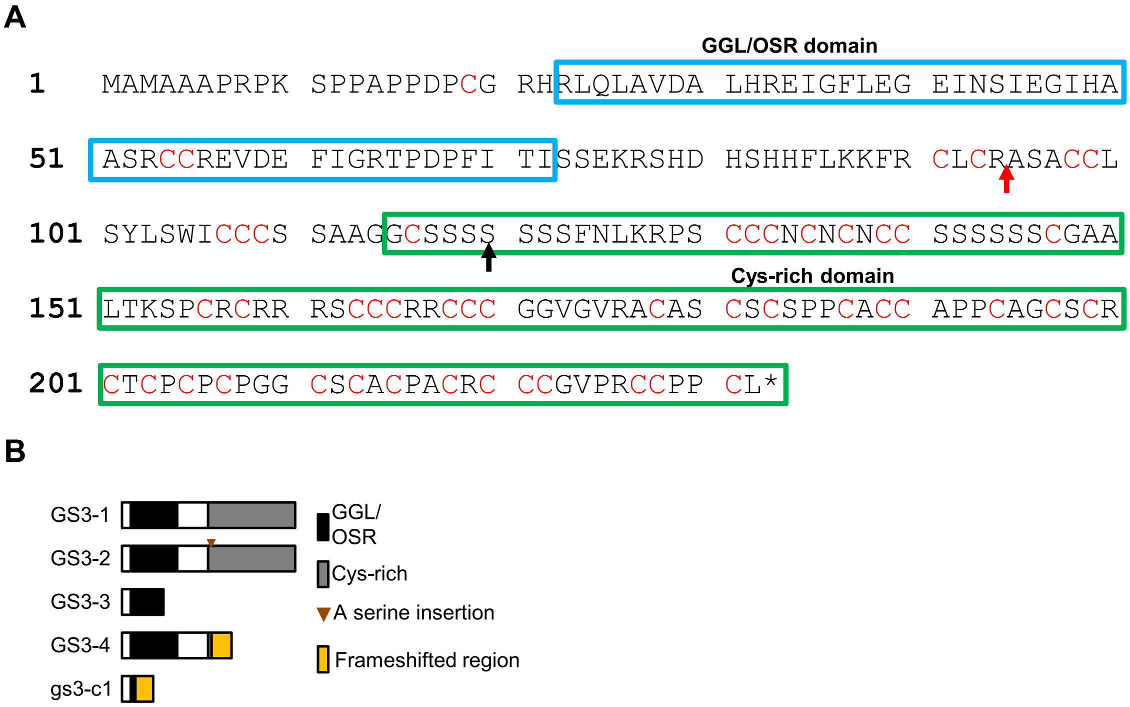

Figure EV1. Amino acids sequence of GS3 protein in ZH11 variety and protein structures of different haplotypes of *GS3*.

(**A**) The amino acid sequence of GS3 from the ZH11 variety, which belongs to the *GS3-2* haplotype, is shown. The GGL/OSR and Cys-rich domains are highlighted in blue and green boxes, respectively, with cysteines labeled in red. The Cys-rich domain comprises 33.9% cysteines (40 out of 118 amino acids), while the C-terminal of GS3 in Fig. 2C, truncated by a red arrow, contains 32.6% cysteines (45 out of 138 amino acids). Additionally, the protein of the *GS3-4* haplotype was frameshifted at the site indicated by the black arrow. (**B**) The protein structures of different haplotypes of *GS3* and *gs3-c1* mutant are shown. Compared to haplotype GS3-1, GS3-2 has a serine insertion without any other variations. GS3-3 encodes a truncated GGL/OSR domain. GS3-4 preserves the GGL/OSR domain but loses most of the Cys-rich domain. gs3-c1 retains a small portion of the GGL/OSR domain and contains a frameshifted region.

© The Author(s)

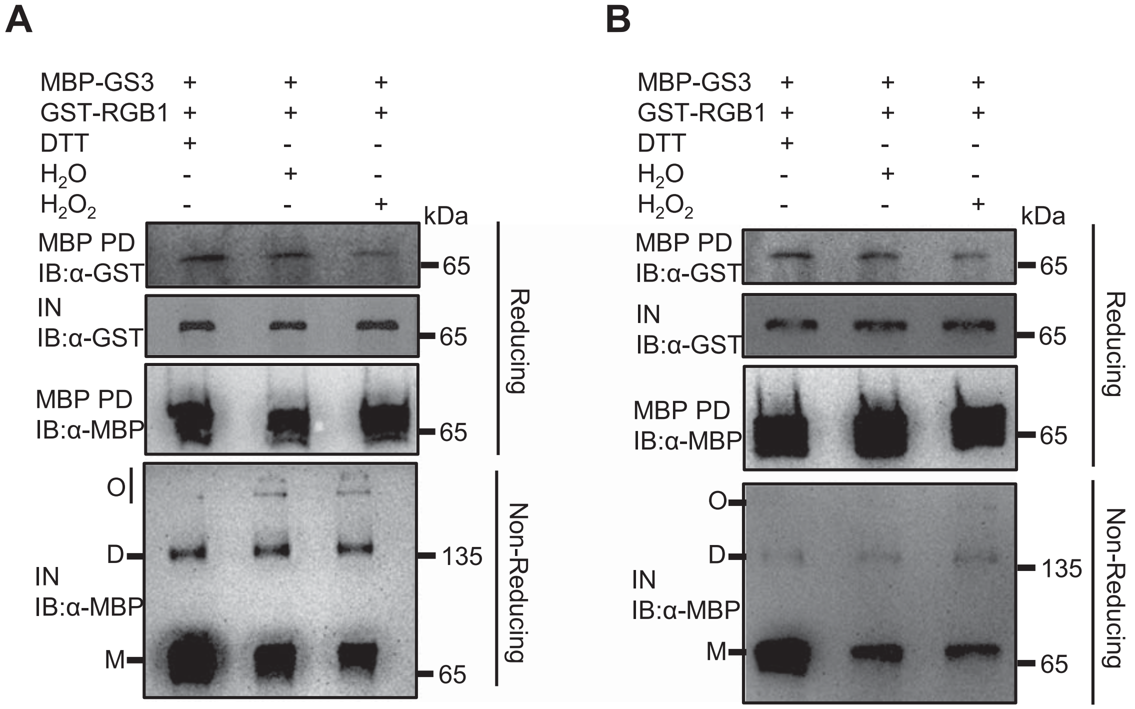

Figure EV2. The oligomerization of GS3 diminishes its interaction ability with RGB1.

(**A**, **B**) Additional two independent repeat experiments of Fig. 3A are shown. M, D, and O in (**A**, **B**) denote monomers, dimers, and oligomers, respectively.

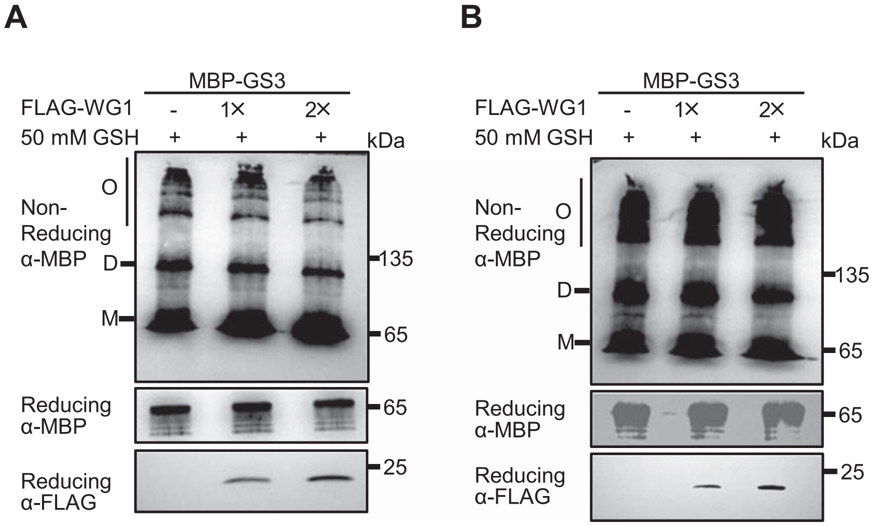

Figure EV3. WG1 can reduce the intermolecular disulfide bonds of GS3 in vitro.

(**A**, **B**) Additional two independent repeat experiments of Fig. 4E are shown. M, D, and O in (**A**, **B**) denote monomers, dimers, and oligomers, respectively.

 © The Author(s)

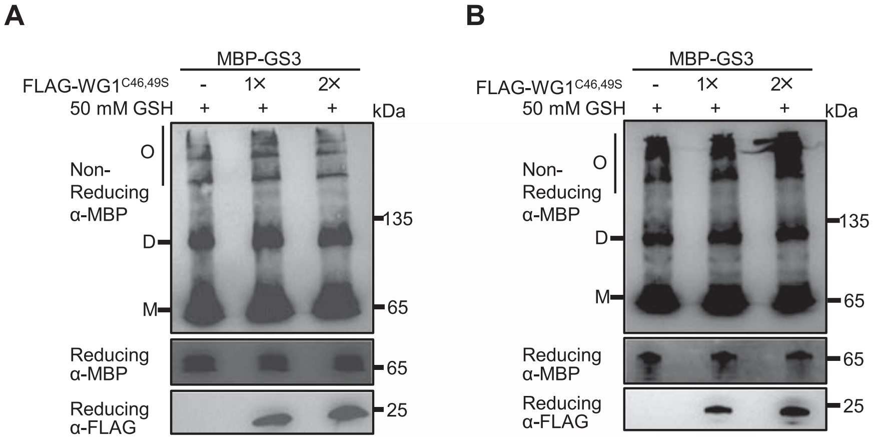

Figure EV4. FLAG-WG1^{C46,49S} loses its ability to reduce the intermolecular disulfide bonds of GS3 in vitro.

(**A**, **B**) Additional two independent repeat experiments of Fig. 4F are shown. M, D, and O in (**A**, **B**) denote monomers, dimers, and oligomers, respectively.

© The Author(s)

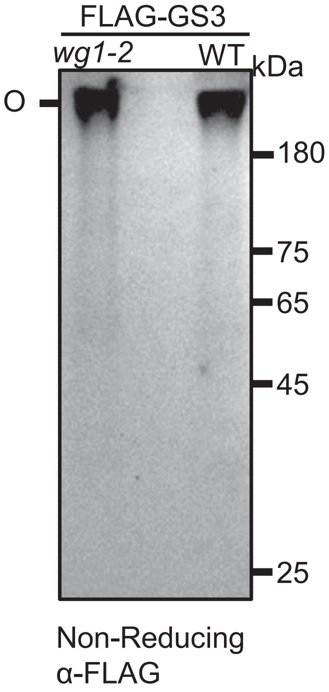

Figure EV5. FLAG-GS3 proteins were detected in an oligomeric state in both *wg1-2* and wild-type protoplasts under non-reducing conditions.

The *pro35S:FLAG-GS3* vector was transiently transformed into both *wg1-2* and wild-type protoplasts. FLAG-GS3 proteins were expressed and separated on a 10% non-reducing SDS-PAGE gel and immunoblotted with an anti-FLAG antibody. O denotes oligomers.

© The Author(s)
